# The Suppressing Effect of Self-Presentation on Social Networks on the Relationship Between Interpersonal Distress and Emotional Experience Among Late Adolescents in a Chinese University: The Moderating Role of Internet Addiction Tendency

**DOI:** 10.3390/bs15030300

**Published:** 2025-03-04

**Authors:** Na Ye, Lu Zhang, Zhiqi You, Hongjuan He

**Affiliations:** 1 Department of Psychology, Wuhan Sports University, Wuhan 430079, China; 2010007@whsu.edu.cn; 2 Department of Social Work, Huazhong Agricultural University, Wuhan 430070, China; zhanglu@mail.hzau.edu.cn (L.Z.); ctyzq@mail.hzau.edu.cn (Z.Y.); 3 Education and Counseling Center for Psychological Health, Zhongnan University of Economics and Law, Wuhan 430073, China

**Keywords:** interpersonal distress, self-presentation on social networks, internet addiction tendency, emotion, college students

## Abstract

This study investigates how self-presentation on social networks suppresses the relationship between interpersonal distress and emotional experience among college students. It also examines the moderating role of internet addiction tendency. A total of 153 college students were surveyed over 8 days. The results showed the following: (1) interpersonal distress is negatively correlated with positive emotions; (2) at both the inter-individual and intra-individual levels, self-presentation on social networks suppresses the negative relationship between interpersonal distress and positive emotions; (3) internet addiction tendency only moderates the relationship between interpersonal distress and self-presentation on social networks at the inter-individual level. These findings suggest that colleges and universities can guide students to use resources on social networks as a means of coping with interpersonal distress.

## 1. Introduction

In daily life, people often encounter trivial troubles, which are usually perceived as stress (Lu & Huang, 2010). One of the most common troubles experienced by college students (Wang, 2015), interpersonal distress, is the pressure people feel during interpersonal interactions, which can easily lead to more negative emotions, such as anxiety and depression, and fewer positive emotions, such as happiness (Guo et al., 2021; Jiao & Mu, 2021; Zhao et al., 2019). The development of the Internet has provided a safer, more flexible, and more convenient channel for people to cope with stress. Individuals can employ various online coping strategies, such as venting emotions and seeking support, to manage the challenges caused by stressful events (van Ingen et al., 2016). College students have a high degree of dependence on social networking sites (SNS) (Tafesse, 2022); therefore, exploring how they can use social networks to mitigate the negative effects of interpersonal distress and achieve adaptive emotional outcomes is a valuable area of research. Self-presentation on social networks refers to people’s efforts to present themselves and influence others on social platforms, to be seen by others as they wish, and is usually divided into positive and authentic self-presentation on social networks (Niu et al., 2015). Both types of self-presentation on social networks help people vent emotions and receive positive feedback or social support, thereby causing more adaptive emotional outcomes (Chen, 2018; Jiang et al., 2022; Liu et al., 2016; Niu et al., 2015). The transactional theory of stress and coping suggests that when people feel that existing resources cannot solve their problems, they will experience stress. In response, they use various coping methods to manage the stress and return to a more balanced, adaptive state. Moreover, people’s coping methods are situational; they choose appropriate coping strategies based on the specific stress they encounter (Lazarus & Folkman, 1984). When people feel interpersonal distress, this stimulates their coping needs, thereby driving them to choose self-presentation on social networks to cope with interpersonal distress and achieve an adaptive emotional state. Because social networks can provide individuals with positive feedback, social support, and other valuable resources, self-presentation on these platforms serves as an effective way to cope with interpersonal distress. In summary, this study posits (H1) that there is a suppressing effect between self-presentation on social networks and adaptive emotions in college students. Specifically, it suggests a positive correlation between interpersonal distress and self-presentation on social networks and a positive correlation between self-presentation on social networks and adaptive emotional experiences.

The transactional theory of stress and coping suggests that people cope with stress resulting from interactions between specific situations and groups of individuals (Lazarus & Folkman, 1987). Therefore, when facing interpersonal distress, people with different characteristics will show different degrees of self-presentation behavior on social networks. Research has found that people with a higher tendency towards internet addiction are more likely to use social networks extensively (Jin et al., 2017), and they can obtain more social support from the network (Ding et al., 2013). It is inferred that people with a higher tendency towards internet addiction are more likely to use social networks to cope when they encounter the stress of interpersonal distress. Therefore, this study assumes (H2) that a higher tendency towards internet addiction will regulate the relationship between interpersonal distress and self-presentation on social networks, thereby regulating the suppressing effect of self-presentation on social networks.

In previous studies that used cross-sectional survey designs to explore the relationship between interpersonal distress and online self-presentation (Li et al., 2018; Qin & Jiang, 2020), the hypotheses proposed based on the transactional theory of stress and coping could not be verified using traditional cross-sectional survey methods (Shao, 2012). This is because interpersonal distress and stress-coping, as measured in traditional cross-sectional studies, often reflect a relatively stable state of life and general psychological tendencies. Additionally, these measurements can be influenced by memory bias. Research has found an inconsistency between overall and daily reports of stress-coping methods (Todd et al., 2004), indicating a specific difference between the stable and situational components of stress-coping. The various stress events faced by college students usually have immediacy (Wang, 2015). Thus, cross-sectional surveys cannot effectively measure and analyze college students’ daily interpersonal distress or the corresponding coping methods (Shao et al., 2019).

Compared to cross-sectional surveys, the daily diary survey can more dynamically measure state variables and avoid measurement errors caused by memory bias to a certain extent, thereby measuring stress, emotions, and coping more meticulously. This type of data also distinguishes between inter-individual and intra-individual effects, allowing for a more precise exploration of the interrelationships between individuals, stress, coping, and emotions (Deng et al., 2015; Shao, 2012). Therefore, this study used a daily diary survey to collect data, examining from a situational perspective whether self-presentation on social networks can improve emotions when college students experience daily interpersonal distress. In other words, it can mask the relationship between interpersonal distress and emotions.

## 2. Methods

### 2.1. Subjects

The study involved 153 undergraduate students from a university in Hubei Province who participated in a 9-day survey. The questionnaire was developed, distributed, and collected through the WJX platform. The specific process is as follows: on the first evening, the participants completed a questionnaire survey that included demographic variables and internet addiction tendencies. From the second day, the participants completed the daily diary survey questionnaire every evening for 8 days. The daily questionnaire included the day’s interpersonal distress, self-presentation on social networks, and emotional experience. The questionnaire was distributed at 21:00 every evening and completed before the participants went to bed. Each questionnaire took about 2 min to complete.

Before analyzing the data, the following steps were taken: First, data that did not meet the requirements for responses or showed obvious tendencies in reaction were eliminated. Second, the diary data were matched and merged with demographic variables and the tendency for internet addiction. Finally, participants with fewer than three valid diary records or missing data on the tendency for internet addiction were excluded.

**Figure 1:**
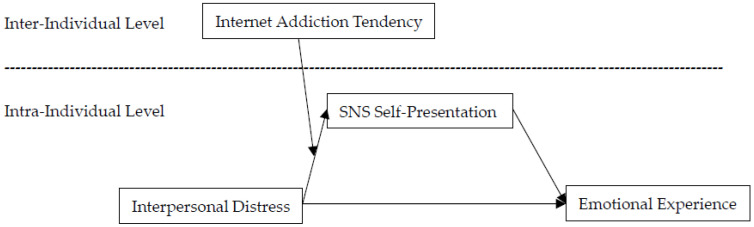
Levels of variable measurement and relationship model.

Ultimately, data were collected from 149 valid participants, who had an average age of 18.3 years (SD = 0.64) and consisted of 101 females and 48 males. These participants provided 1046 valid data points, resulting in a response rate of 87.8%. The structure of the measurement levels for each variable is shown in Figure 1.

### 2.2. Measurement Tools (Appendix A)

The·tools·used in·this·study·were summarized in Appendix A.

#### 2.2.1. Positive and Negative Experience Scale

The Positive and Negative Experience Scale developed by Diener et al. (2010) was used. It includes two subscales: positive emotions and negative experiences, each consisting of 6 items. Responses were measured using a 5-point Likert scale (1 = none, 5 = always). In this study, the Cronbach’s alpha coefficients for the positive experience and negative experience subscales measured over 8 days were between 0.93–0.97 and 0.88–0.97, respectively.

#### 2.2.2. Daily Troubles Questionnaire for College Students

The 3 items with the highest scores from the interpersonal stress subscale of the Daily Stress Questionnaire for College Students, developed by Lu (2010), were selected to measure each day’s interpersonal distress. A 5-point Likert scale (0 = no impact, 4 = great impact) was used. The Cronbach’s alpha coefficients measured over 8 days were between 0.86 and 0.91.

#### 2.2.3. Self-Presentation on Social Networks Questionnaire

Based on the items and rating methods of existing self-presentation on social networks questionnaires (Niu et al., 2015), 3 items were developed to measure the participants’ activity on online social platforms each day. A 6-point Likert scale (0 = none, 5 = a lot) was used. The Cronbach’s alpha coefficients measured over 8 days ranged from 0.79 to 0.90.

#### 2.2.4. Internet Addiction Diagnostic Questionnaire

The Internet Addiction Diagnostic Questionnaire, developed by Young (1998), was used in this study. It consists of 8 items in total. A 6-point Likert scale (1 = not at all, 6 = completely) was used to measure the participants’ tendency towards internet addiction (You et al., 2020). The Cronbach’s alpha coefficient in this study was 0.85.

In this study, the level of each variable was measured using the average score of items, with higher scores indicating higher levels of the variable.

### 2.3. Statistical Methods

The data collected in this study have a two-level structure (see Figure 1). Positive emotions, negative emotions, interpersonal distress, and self-presentation on social networks were measured at the intra-individual level, with ICC(1) values of 0.61, 0.55, 0.62, and 0.54, respectively, and ICC(2) values of 0.92, 0.89, 0.92, and 0.89, respectively. The tendency for internet addiction was measured at the inter-individual level. Therefore, this study used the MLmed macro program in IMB SPSS 25 for multilevel analysis to verify the moderated mediation model across the levels. During the analysis, interpersonal distress (X) and self-presentation on social networks (M) were group mean-centered and included in the first-level equation, with their group means added to the second-level equation to separate intra-individual and inter-individual effects. The tendency for internet addiction was included in the second-level equation to predict the slope of interpersonal distress, capturing the intra-individual level moderation effect. Additionally, the interaction term between the tendency for internet addiction and the group mean of interpersonal distress was included in the second-level equation to predict the intercept, reflecting the inter-individual level moderation effect. This allows us to separate the intra-individual and inter-individual effects to more comprehensively and accurately estimate the relationships between variables.

## 3. Results

### 3.1. Descriptive Analysis

The results of the correlation analysis of various variables are shown in Table 1. Except for the non-correlation between self-presentation on social networks and negative emotions at the inter-individual level, there is a correlation between the independent, dependent, and mediating variables.

### 3.2. Multilevel Moderated Indirect Effect Analysis

Analyses were conducted using positive and negative emotions as dependent variables in a multilevel indirect model, and the results presented in Table 2. At both the inter-individual and intra-individual levels, interpersonal distress effectively predicts self-presentation on social networks positively and positive emotions negatively. Self-presentation on social networks effectively predicts positive emotions. Due to the inconsistent directions of the direct and indirect paths, self-presentation on social networks exhibits a suppressing effect on interpersonal distress and positive emotions at both levels. The suppressing effect of self-presentation on social networks is 0.04 (SE = 0.02, *p* = 0.05, 95%CI = [0.01, 0.09]) at the inter-individual level and 0.01 (SE = 0.003, *p* = 0.059, 95%CI = [0.001, 0.01]) at the intra-individual level. Regardless of the inter-individual or intra-individual levels, there is a positive correlation between interpersonal distress and negative emotions. Still, since self-presentation on social networks is not related to negative emotions, it has no indirect effect on the relationship between interpersonal distress and negative emotions.

Internet addiction tendency was included in the model as a moderating variable, after being mean-centered, to regulate the relationship between interpersonal distress and self-presentation on social networks. Since self-presentation on social networks has no indirect effect on interpersonal distress or negative emotions, only the moderating effect of internet addiction tendency in the model with positive emotions as the dependent variable was analyzed. The results of the multilevel moderated indirect model analysis show that the tendency for internet addiction does not have a moderating effect at the intra-individual level. However, it does moderate the positive relationship between interpersonal distress and self-presentation on social networks at the inter-individual level, with a moderating effect of 0.21 (SE = 0.09, *p* < 0.05, 95% CI = [0.02, 0.40]). This suggests that internet addiction tendency strengthens the correlation between interpersonal distress and self-presentation on social networks.

The moderating effect of internet addiction tendency on the indirect relationship between interpersonal distress, self-presentation on social networks, and positive emotions is significant only at the inter-individual level, with a value of 0.033 (95% CI = [0.002, 0.079]). When the internet addiction tendency was set to one standard deviation above and below the mean for indirect effect analysis, it was found that under conditions of high internet addiction tendency, the suppressing effect of self-presentation on social networks at the inter-individual level is significant, at 0.07 (SE = 0.03, *p* < 0.05, 95% CI = [0.02, 0.14]); under conditions of low internet addiction tendency, the suppressing effect of self-presentation on social networks at the inter-individual level is not significant, at 0.01 (SE = 0.02, *p* = 0.56, 95% CI = [−0.02, 0.06]).

## 4. Discussion

Previous researchers have analyzed the relationship between interpersonal distress and self-presentation on social networks using cross-sectional survey data based on theories such as social compensation and impression management (Li et al., 2018; Qin & Jiang, 2020). This study, based on the transactional theory of stress and coping, examines the relationship between situational interpersonal distress and self-presentation on social networks. Due to its analysis of daily diary data collected over 8 days, the research broadens the existing knowledge in this area. This study found that regardless of the inter-individual or intra-individual level, there is a positive correlation between daily interpersonal distress and self-presentation on social networks in college students. Although self-presentation on social networks is not related to negative emotions, it is positively related to positive emotions. Since interpersonal distress is negatively correlated with positive emotions, self-presentation on social networks partially mitigates this relationship between interpersonal distress and positive emotions, suggesting that it plays a role in stress-coping strategies. The correlation at the inter-individual level reflects the relationship between each independent variable and the intercept of the equation at the intra-individual level, which can be seen as a relationship like the trait measurement of independent and dependent variables (Hayes & Rockwood, 2020). The results of the indirect model at the inter-individual level suggest that individuals who experience more interpersonal distress are likely to engage in more self-presentation on social networks. This increased self-presentation helps them experience more positive emotions, which in turn helps counteract the negative impact of interpersonal distress on their overall mood. The correlation at the intra-individual level reflects the prediction of the daily level of the dependent variable by the daily level of the independent variable that is more or less than the general situation (mean), which can be seen as the influence of the daily fluctuation of each independent variable on the dependent variable (Hayes & Rockwood, 2020). The results of the indirect model at the intra-individual level indicate that when college students experience more interpersonal distress than usual, they will have fewer positive emotions and will show more self-presentation on social networks. More self-presentation on social networks than usual is related to more positive emotional experiences. It can be seen that self-presentation on social networks can buffer the reduction of positive emotions caused by facing more interpersonal distress than usual.

According to the transactional theory of stress and coping (Lazarus & Folkman, 1984), the above results indicate that self-presentation on social networks meets the need of college students to cope with interpersonal distress and can be regarded as an effective strategy to cope with daily interpersonal distress. Self-presentation on social networks not only provides college students with a safer opportunity to vent emotions, but also provides opportunities to obtain instrumental or emotional support (van Ingen et al., 2016; Jiang et al., 2022; Liu et al., 2016; Niu et al., 2015), and both emotional venting and support are resources for college students to cope with interpersonal distress.

This study also found that a tendency towards internet addiction can only regulate the relationship between interpersonal distress and self-presentation on social networks at the inter-individual level. The stronger the tendency towards internet addiction, the stronger the positive correlation between interpersonal distress and self-presentation on social networks; the weaker the tendency towards internet addiction, the less relevant the two become. This indicates that people with a strong tendency towards internet addiction, if often troubled by interpersonal problems, are more inclined to use self-presentation on social networks for stress-coping. In contrast, those with a weak tendency towards internet addiction do not use self-presentation on social networks as a general way to cope with interpersonal distress. This result is also consistent with the transactional theory of stress and coping, which suggests that people choose coping strategies based on how they assess both the situation and their abilities (Lazarus & Folkman, 1987). In addition, the moderation effect of a tendency towards internet addiction is not significant at the intra-individual level, which indicates that when encountering interpersonal problems beyond the general level, regardless of their tendency towards internet addiction, college students are more willing to cope through self-presentation on social networks. Self-presentation on social networks is a common way for college students to cope with prominent interpersonal distress.

This study does have certain limitations. In terms of research design, although the diary method used in this study can measure more detailed and situational interpersonal distress, self-presentation on social networks, and emotional states, the measurement time points are all the same, making it impossible to prove causal relationships between variables. According to Gangemi et al. (2021), negative emotions can lead people to negatively evaluate their lives. Therefore, individuals with more negative emotions are likely to perceive greater interpersonal distress, potentially creating a vicious cycle. Future research could employ experimental methods or stagger the measurement time points of different variables—for example, measuring interpersonal distress during the day in the evening and assessing social media self-presentation and emotional states before bedtime to further verify the causal mechanisms.

Regarding the research sample, the participants in this study were predominantly first-year students, with a significant overrepresentation of female participants compared to males. This demographic skew limits the generalizability of the findings to broader college student populations. Consequently, future studies should aim to recruit a more diverse and representative sample, encompassing students across different academic years and ensuring a balanced gender distribution to enhance the external validity of the results.

Regarding the research variables, this study solely assessed the general state of self-presentation on social networks, overlooking the intricate nature of self-presentation on social networks. Research has demonstrated that authentic self-presentation can bolster individuals’ positive emotions (Bailey et al., 2020). Conversely, other studies have revealed that some forms of self-presentation on social networks may prompt adolescents to seek more feedback and engage in upward social comparisons, while simultaneously heightening their susceptibility to negative evaluations, consequently impairing their mental health (Nesi & Prinstein, 2015; Evangelou et al., 2024). It is apparent that the influence of self-presentation on adolescent development is complex and multifaceted. Hence, future research needs to meticulously examine the mechanisms through which different characteristics of online self-presentation influence adolescents’ ability to cope with real-life stress.

The findings of this study suggest that, in the information age, social networks serve as a critical resource for adolescents to navigate prominent interpersonal challenges and manage emotional stress. Consequently, educational institutions should consider developing well-designed and secure social network platforms, such as online counseling systems, to harness the positive potential of these networks. Such platforms would provide college students with opportunities for self-expression and constructive feedback, thereby aiding them in addressing everyday emotional difficulties. Nevertheless, it is imperative for schools to actively minimize the adverse effects of social networks on adolescent development within these facilities. Measures should include preventing inappropriate negative feedback, curbing excessive reliance on the platform, and guiding adolescents to build robust offline support networks. Furthermore, these platforms should be utilized to foster adolescents’ interpersonal skills and enhance their emotional regulation capabilities.

## 5. Conclusions

These research findings show a significant negative correlation between interpersonal distress and positive emotions. However, self-presentation on social networks helps suppress this negative effect, both at the between-person and within-person levels. Notably, a tendency for internet addiction moderates the relationship between interpersonal distress and social media self-presentation only at the between-person level. No significant moderating effect is observed at the within-person level.

## Figures and Tables

**Table 1:** Basic descriptive statistics and correlation analysis results.

	$\bar{x}$ ± *s*	1	2	3	4
1 Positive Emotions	3.46 ± 0.25	1	−0.43 ***	−0.15 ***	0.16 ***
2 Negative Emotions	1.95 ± 0.23	−0.42 ***	1	0.41 ***	0.06 *
3 Interpersonal Distress	1.21 ± 0.85	−0.14 †	0.49 ***	1	0.16 ***
4 Self-Presentation on Social Networks	1.31 ± 0.93	0.18 *	0.10	0.24 **	1
5 Internet Addiction Tendency	2.66 ± 0.88	−0.14 †	0.28 ***	0.15 †	0.16 †

Note: † *p* < 0.10, * *p* < 0.05, ** *p* < 0.01, *** *p* < 0.001, the same below. Means and standard deviations are the results of the inter-individual level analysis (N = 149); below the diagonal are inter-individual correlation coefficients (N = 149), and above the diagonal are intra-individual correlation coefficients (N = 1046).

**Table 2:** Multilevel indirect model analysis results.

	Dependent Variable: SNS Self-Presentation			Dependent Variable: Positive Emotions			Dependent Variable: Negative Emotions		
	*B*	*SE*	*95%IC*	*B*	*SE*	*95%IC*	*B*	*SE*	*95%IC*
Intra-Individual									
Constant Term	1.00 ***	0.13	[0.75, 1.26]	3.43 ***	0.11	[3.23, 3.64]	1.57 ***	0.08	[1.40, 1.74]
Interpersonal Distress	0.10 *	0.04	[0.02, 0.18]	−0.13 ***	0.03	[–0.18, −0.08]	0.23 ***	0.02	[0.18, 0.27]
SNS Self-Presentation				0.07 ***	0.02	[0.03, 0.11]	0.02	0.02	[−0.01, 0.06]
Inter-Individual									[3.23, 3.64]
Interpersonal Distress	0.25 **	0.09	[0.08, 0.42]	−0.15 *	0.06	[−0.27, −0.03]	0.32 ***	0.05	[0.23, 0.42]
SNS Self-Presentation				0.16 **	0.06	[0.05, 0.27]	−0.01	0.05	[−0.10, 0.08]
Residual	0.64 ***	0.03	[0.58, 0.70]	0.23 ***	0.01	[0.21, 0.25]	0.21 ***	0.01	[0.19, 0.23]

Note. * *p* < 0.05; ** *p* < 0.01; *** *p* < 0.001.

## Data Availability

All data generated or analyzed during this study are available from the corresponding author upon reasonable request.

## Appendix A

Internet Addiction Tendency

Below are some descriptions of internet use. Please judge the degree to which these descriptions match your personal situation and choose the appropriate score. The higher the score, the more it matches your actual situation.

Completely Disagree	Somewhat Disagree	Slightly Disagree	Slightly Agree	Somewhat Agree	Completely Agree
1. Do you feel preoccupied with the Internet (think about previous on-line activity or anticipate next on-line session)? 2. Do you feel the need to use the Internet with increasing amounts of time in order to achieve satisfaction? 3. Have you repeatedly made unsuccessful efforts to control, cut back, or stop internet use? 4. Do you feel restless, moody, depressed, or irritable when attempting to cut down or stop internet use? 5. Do you stay on-line longer than originally intended? 6. Have you jeopardized or risked the loss of a significant relationship, job, educational, or career opportunity because of the Internet? 7. Have you lied to family members, a therapist, or others to conceal the extent of involvement with the Internet? 8. Do you use the Internet as a way of escaping from problems or of relieving a dysphoric mood (e.g., feelings of helplessness, guilt, anxiety, depression)?					

Interpersonal Distress

The following table lists some events that may happen in students’ lives. Please choose the appropriate score to indicate whether these events have affected your life today, with 0 being no impact and 4 being great. The higher the score, the greater the impact.

0 No Impact at All	1 A Slight Impact	2 A Moderate Impact	3 A Significant Impact	4 A Great Deal of Impact
1. Disappointing with friends 2. Being rejected by others 3. Being misunderstood or wronged				

Self-presentation on social network

Today, please rate how often the following behaviors occur in you by choosing the appropriate number. The higher the level, the more the behavior is exhibited.

Rarely	Less often	Occasionally	Fairly often	Very often
1. Posting your status or opinions on social networking platforms (such as Qzone, WeChat Moments, Weibo, etc.). 2. Posting positive status on social networking platforms (such as Qzone, WeChat Moments, Weibo, etc.). 3. Posting negative status on social networking platforms (such as Qzone, WeChat Moments, Weibo, etc.).				

Emotional Experience

The following is a list of emotional descriptive words. Please review your experiences today and evaluate your emotional experiences in these aspects today. The higher the score, the more frequently the emotion is experienced.

Never	Occasionally	Sometimes	Often	Always
1. Positive 2. Negative 3. Good 4. Bad 5. Pleasant 6. Unpleasant 7. Happy 8. Sad 9. Fearful 10. Joyful 11. Angry 12. Satisfied				
